# Supplementary figures and images for: EIF4A3 serves as a prognostic and immunosuppressive microenvironment factor and inhibits cell apoptosis in bladder cancer
Source: PeerJ. 2023 May 8;11:e15309. doi: 10.7717/peerj.15309 (PMC10174062; doi:10.7717/peerj.15309)

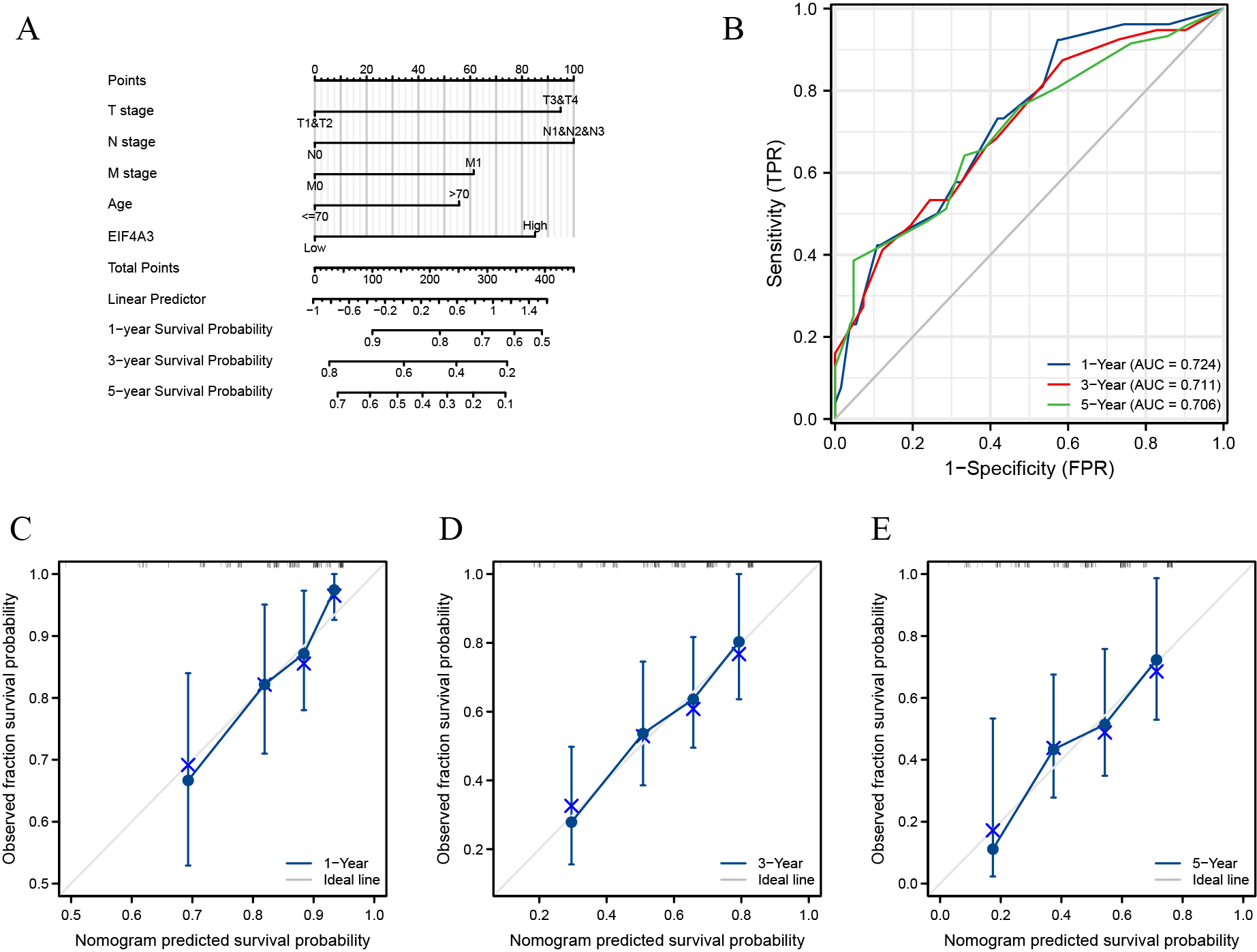

Supplement: Figure S1 — (A) The predicted 1, 3, and 5-year OS for BLAC patients by the nomogram. (B) The 1, 3, and 5-year AUC values of the nomogram. (C-E) The 1-year, 3-year, and 5-year calibration plots of the nomogram respectively. [file peerj-11-15309-s001.png]

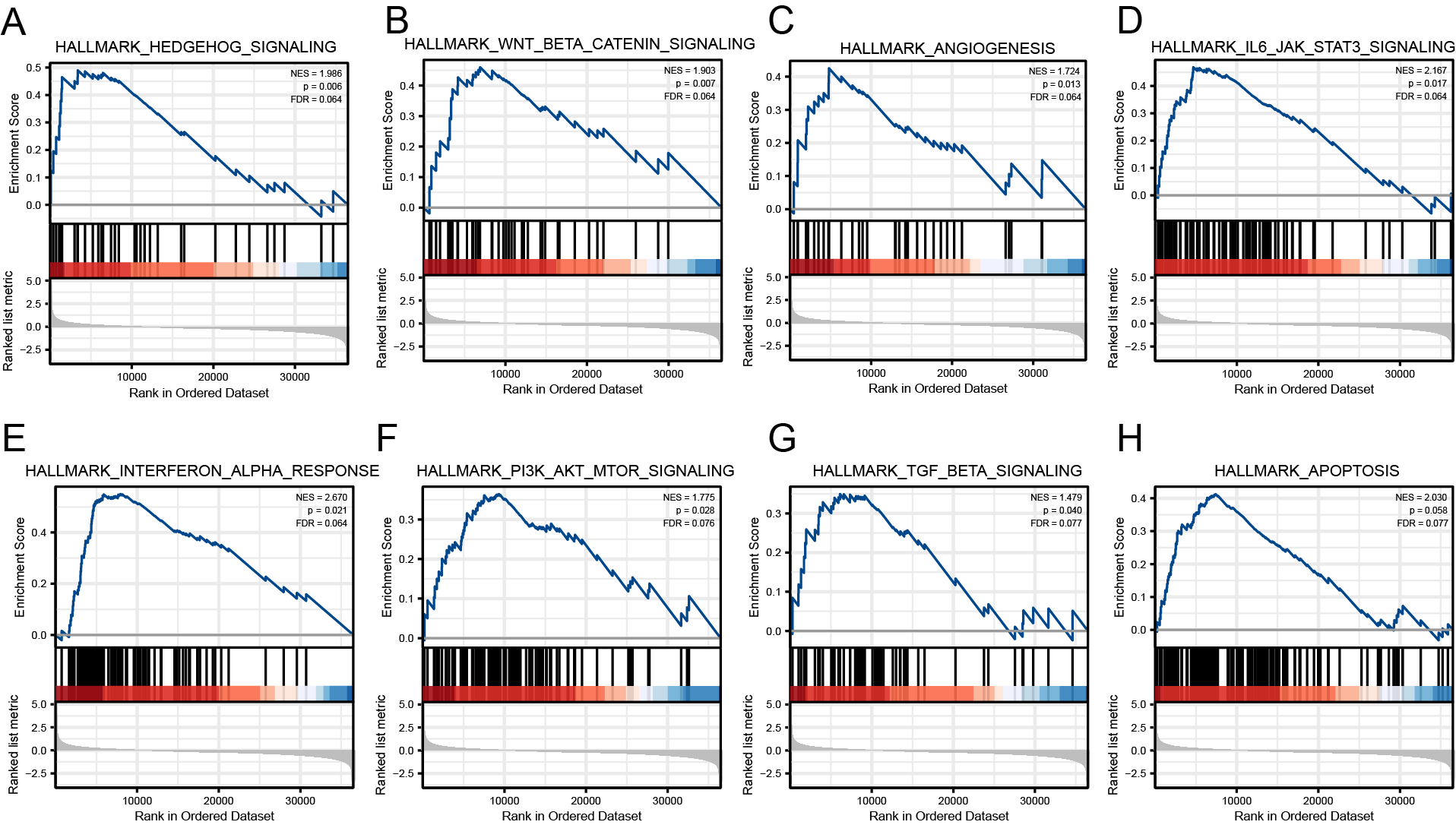

Supplement: Figure S2 — (A-H) Hedgehog, Wnt- β-catenin, Angiogenesis, IL6-JAK-STAT3, Interferon- α response, PI3K-AKT-mTOR, TGF- β, and Apoptosis. [file peerj-11-15309-s002.png]
